# Does country of resettlement influence the risk of suicide in refugees? A case-control study in Sweden and Norway

**DOI:** 10.1192/j.eurpsy.2022.338

**Published:** 2022-09-01

**Authors:** R. Amin, E. Mittendorfer-Rutz, L. Mehlum, B. Runeson, M. Helgesson, P. Tinghög, E. Björkenstam, E. Holmes, P. Qin

**Affiliations:** 1 Karolinska Institutet, Clinical Neuroscience, solna, Sweden; 2 University of Oslo, National Centre For Suicide Research And Prevention, Oslo, Norway; 3 University of Oslo, National Centre For Suicide Research And Prevention, solna, Sweden; 4 Swedish Red Cross University College, Health Sciences, Huddinge, Sweden; 5 Karolinska Institutet, Division Of Psychology, Department Of Clinical Neuroscience, solna, Sweden

**Keywords:** Suicide, Country of birth, Duration of residence, Refugee

## Abstract

**Introduction:**

Little is known regarding how the risk of suicide in refugees relates to their host country. Specifically, to what extent, inter-country differences in structural factors between the host countries may explain the association between refugee status and subsequent suicide is lacking in previous literature.

**Objectives:**

We aimed to investigate the risk of suicide among refugees in Sweden and Norway according to their sex, age, region/country of birth and duration of residence.

**Methods:**

Each suicide case between the age of 18-64 years during 1998 and 2018 (17,572 and 9,443 cases in Sweden and Norway, respectively) was matched with up to 20 population-based controls, by sex and age. Multivariate-adjusted conditional logistic regression models yielding adjusted odds ratios (aORs) with 95% confidence intervals (95% CI) were used to test the association between refugee status and suicide.

**Results:**

The aORs for suicide in refugees in Sweden and Norway were 0.5 (95% CI: 0.5-0.6) and 0.3 (95% CI: 0.3-0.4), compared with the Swedish-born and Norwegian-born individuals, respectively. Stratification by region/country of birth showed similar statistically significant lower odds for most refugee groups in both host countries except for refugees from Eritrea (aOR 1.0, 95% CI: 0.7-1.6) in Sweden. The risk of suicide did not vary much across refugee groups by their duration of residence, sex and age.

**Conclusions:**

The findings of almost similar suicide mortality advantages among refugees in two host countries may suggest that resiliency and culture/religion-bound attitudes could be more influential for suicide risk among refugees than other post-migration environmental and structural factors in the host country.

**Disclosure:**

No significant relationships.

